# What Causes Variety and Modifications in the Character of Dental Caries

**Published:** 1892-02

**Authors:** J. G. Junkerman


### What Causes Variety and Modifications in the Character of Dental Caries.

BY J. G. JUNKERMAN, M.D., D.D.S

Read before the Ohio State Dental Society, December 3, 1891.

A paper upon this subject can scarcely possess the character of originality, but will subserve the purpose more of bringing out, before such a body as this, the points for discussion.

There are no longer grounds for disagreement among the profession upon the causes of dental caries, and knowing the causes of the disease a study of the character of the agents producing it will reveal, to a considerable extent, the cause of variety and modifications in the character of dental caries. We may sum up the cause of dental caries as acids and fungi. Although the investigations of Miller, of Berlin, have confirmed this hypothesis, yet in the minds of many practitioners this conclusion has been accepted for many years past. The chief agents, without which there would be no dental caries, being acids and fungi, the character of the process of the caries must depend upon the character of the acids and fungi producing it. The process of dental caries consists of the decomposition, or the solution of the inorganic matter of the tooth by the acids, and the decomposition by the fungi of the organic structure of the tooth. Assuming that the fungi are alike in all cases, we know the acids to be different and upon the character of these acids will depend the character of the caries. The character of the carious process will differ as differs the degree of concentration of the acid, the quantity and the degree of persistence with which it remains at the seat of the disease.

The character of dental caries will be influenced much also depending upon the order in which decomposition of the tooth structures takes place. If the fungi decompose their part of the structure first we will have a caries of rather a hard consistency; a leathery tending to a softer consistency of the caries will result if the acid dissolves or decomposes the calcareous matter previous to the action of the fungi. There can be no doubt that the consistency of dental caries is dependent upon the time of action of the acid and the fungi.

Quality of tooth structure modifies the character of dental caries. Whatever the character of the agent may be, whether concentrated or diluted, whether persistent or intermittent, or in whatever stage of caries the case may be, the well organized or perfect structured tooth will show a slower progress of disease than the tooth of weak structure. To further elucidate this principle and to present it to the mind in the extreme: when the chemist wishes to dissolve a substance in an acid, or any other menstrum, he pulverizes the substance to produce rapid solution. This comminution of the substance brings about a condition whereby a larger extent of surface is presented for contact with the menstrum, and it is a known fact that rapidity of solution is in proportion to the. extent of surface. This principle may be applied to the process of dental caries. If the enamel prisms, or dental tubuli, are loose in structure there is a greater extent of surface, and rapidity of caries is increased, and though imperfection of structure may be invisible to the naked eye, this ever watchful agent of dental caries has its headlights and reflectors into all the alleys and byways of imperfect development, and is always ready with its regiments of sharp-toothed soldiers to take up its camping ground upon any unoccupied tracts.

Another circumstance which modifies the character of dental caries rests in the fact of the difference in chemical structure between the enamel and dentine. The lime salts of enamel are in excess of those found in dentine, so that an acid may be found strong enough, and in sufficient quantity to dissolve dentine, while the enamel will be little or none at all affected. Dental caries arising from such a condition would find explanation in cavities of considerable depth and small external openings. Such cavities would be the result of weak acids and long-continued action. Caries with wide external openings and superficial in character would result from sudden strong acid reaction and short duration. In both these cases the character of the dental caries is not due to the character of the agent producing it, but is most likely due to the difference in chemical structure of the enamel and dentine. A condition which characterizes the process of dental caries is sensitiveness or a lack of sensitiveness. Sensitiveness accompanies that process of dental caries where the acid of the disease operates more rapidly than the fungi. Such are the soft varieties of caries. It does not then follow that all soft caries are sensitive, for to produce this condition the process of the disease must be such that the dental tubuli are left almost so, or entirely intact. A lack of sensibility will attend that process of dental caries where the fungi are the most rapid in their action, destroying the organic tissue or dental tubuli and leaving the lime salts, producing the hard variety of caries. From these principles we would deduce the conclusion that the very soft and the very hard varieties of dental caries would be free of sensibility, and that we could look for sensitiveness in the semi-hard varieties of dental caries.

Color is another characteristic feature of dental caries. It varies from white to black. The responsibility for the color rests much upon the nature of the agent, but not entirely so, as deposits upon the teeth and the habits of the individual in general, have some influence upon the subject. For example: the tobacco chewer may be afflicted with the white variety of caries, and yet the color may be black from the infiltration of nicotine. The black variety of caries may lose its characteristic color from the introduction into the oral cavity of a variety of decolorizers.

There passes before the eyes of the practitioner a daily panorama of varying changes in the character of dental caries; there is slow caries and rapid caries; caries deep in character with small external opening; caries with large external opening and superficial in character; caries hard in consistency and caries soft and without form; caries that is painful and that which lacks sensibility; caries with color and caries without color. These qualities combined give character to dental caries and the absence or presence of one or more of them is what produces variety and modifications in the character of the process of dental caries.

DISCUSSION ON DR. JUNGERMAN'S PAPER.

*Dr. J. R. Callahan*, Cincinnati. I would like to ask Dr. Junkerman a question. He referred to the fungi, saying that he assumed that those present were all alike instead of saying they were alike.

*Dr. G. S. Junkerman*:—I said I assumed they were all alike, for we do not know sufficient at present to distinguish between the various fungi and the degree or kind of caries that arises from them; that will have to be discovered by further investigation. We know more about the acids than we do about the fungi and their action.

*Dr. Geo. H. Wilson*, Painesville. There was one remark made by the essayist that seems to me a little broad and one which we might take exception to if I understood him correctly, and that is, cavities having a small opening would be of slow decay. It seems in practice we see the most rapid form of decay in that condition in which, the dentine is destroyed more rapidly than the enamel, consequently we have rapid decay on the inside, just a mere shell of the tooth. When we break into such a cavity we may have a bad case on hand. I was very much pleased with the paper, but I think we sometimes make statements that are a little broad.

*Dr. C. M. Wright*:—I have very little to say. I was pleased with the manner of presentation of the paper and its conciseness, and consider it a valuable one for our archives. I was somewhat amused at the beginning of the paper by the statement of the gentleman that there is no dispute in the dental profession as regards the etiology of dental caries. If he had been in the room last night and heard the discussion between Professors Taft and Smith he might have changed his paper somewhat. I have no bones to pick in regard to the paper, nor is there any thing that I can quarrel with, consequently I am out of my element.

*Dr. H. A. Smith*:—I regret I was not present when the paper was read. I missed it, therefore I can not discuss it, except the point raised a moment ago by a gentleman in reference to why we had in some conditions of caries quite a spreading variety, and the other varieties had penetrated directly in the shortest direction towards the pulp. If the dentine is of uniform density, if it is perfectly alike throughout the entire structure, it is difficult to explain why caries assumes this form, while in other cases it is directly in a straight line or in the direction towards the pulp. May we not assume that when we have spreading caries the dentine is not homogeneous; that we have defective territories or inter-zonal spaces, as they are called. When the active cause reaches that defective territory it has modified or changed the direction of the progress of the disease. Would that be an explanation of all these cases? We find a variety of caries spreading under the enamel, the inter-zonal space, which is more susceptible, more predisposed to caries than dentine proper in the body of a tooth. I would like to have the gentleman refer to these anomalies, to give us his explanation of them since they are not clear in my own mind.

At a recent meeting of the Pathological Society, Dr. Leonard Guthrie showed a toad whose death had been caused by Larvæ of Blow Flies having attacked its mouth and nostrils. The nostrils formed one large cavity, separated only by a thin septum of skin anteriorly; both eyes were collapsed, and their empty tunics lay in the cavity of the mouth, whilst the whole of the soft palate had been devoured by the larvæ, leaving the bones picked bare. Between three and four dozen larvæ were removed after the death of the toad. It was well known that toads and frogs were subject to such ravages, but until the present time no attempt to identify the larvæ had been successful. Dr. T. S. Cobbold had referred to the subject in the *Veterinarian* for 1880, but had been unable to name the maggots. Professor Brauer, of Vienna, had kindly examined the larvæ now exhibited, and had pronounced them to be of the genus "Calliphoræ." The species was indeterminate, but was probably *Erythro-cephala magna* or *Vomitina.* It was difficult to explain why the mouth and nostrils of batrachians were always the sole parts attacked. It was obviously impossible for the fly to lay its eggs directly in the toad's nostrils, and the probable explanation was that the eggs were laid in the toad's mouth, whilst a pregnant fly was being swallowed. It was stated that the number of toads had been greatly reduced by similar attacks of such larvæ.

In the discussion—Dr. Beaven Rake said that in tropical countries maggots in the nose and in the external auditory meatus were very common, especially in lepers, and also in persons in very different stations in life. He mentioned the case of a man who was stung by a fly, and later a large larva was expressed from a swelling which had formed at the spot.—(*British Journal of Dental Science*).

